# Partial Obstruction of the Endotracheal Tube by the Plastic Coating Sheared from a Stylet

**DOI:** 10.1155/2016/4373207

**Published:** 2016-02-16

**Authors:** Anirudha Das, Shwetha Chagalamarri, Kim Saridakis

**Affiliations:** Metro Health Medical Center, Cleveland, OH 44109, USA

## Abstract

A preterm with gestational age of 24 weeks was intubated at day of life 16. The intubation was done in a routine manner with the use of a stylet. It took a significant effort from the clinician to pull the stylet out after intubation. After intubation the respiratory status of the neonate deteriorated requiring significantly greater support. When ventilating and oxygenating the infant was getting progressively difficult, the decision was made to change the endotracheal tube (ETT). The cause for deterioration of respiratory status was then determined to be a sheared piece of plastic from the sheath of the stylet which was lodged in the lumen of the ETT. After removal of the plastic particle, the condition of the infant improved significantly.

## 1. Introduction

Equipment failure is one of the considerations when acute deterioration of respiratory status is encountered in a Neonatal Intensive Care Unit (NICU) setting. Blocked endotracheal tube (ETT) is a common reason for failure of ventilation or oxygenation. It is common for the ETT to be blocked by secretions but we present a case where a sheared-off piece of plastic tube from the stylet during the process of intubation leads to the blockage of the lumen of the ETT. To the best of our knowledge, this rare occurrence has been reported no more than four times in the past.

## 2. The Case

A premature female newborn with a gestation of 24 weeks and 4 days and a birth weight of 530 grams was reintubated at day 16 of life due to accidental extubation. The infant was intubated and ventilated since birth for respiratory failure. The usual technique of intubating in the Neonatal Intensive Care Unit (NICU) at this Center was to use a stylet inserted into the endotracheal tube (ETT) at the time of intubation.

In this case the ETT used was a 2.5-millimeter internal diameter, uncuffed, single use tube. The stylet was a 6-French size sterile disposable lubricated intubating stylet. It was made in China and distributed by Cardinal Health, Waukegan, Illinois. The manufacture date on the label was 10/2013 and the expiry date was 09/2018, usable at the time of the incident. It was a plastic sheathed, malleable aluminum stylet. The plastic sheath extended beyond the tip of the metal stylet to minimize the risk of tracheal trauma.

The usual protocols of the unit were followed at the time of intubation. The unusual observation during the process was that the physician had to use more than usual force while pulling the stylet out of the ETT after intubation.

Over the next several hours the child deteriorated significantly in terms of respiratory status. The ventilator setting had to be increased to manage persistent desaturations and retained carbon-dioxide (CO_2_). On clinical examination there was diminished chest rise and faint breath sounds. A chest X-ray showed areas of atelectasis of the lungs bilaterally. The child was switched to an oscillator to improve the ventilation but hardly any “wiggle” could be observed over the chest, in spite of increasing the amplitude. Finally, a decision was made to reintubate the infant. When the ETT was pulled out, the reason behind the deteriorating respiratory status was established. The plastic tip of the stylet had been sheared off from the aluminum core and was lodged on the distal end of the ETT hardly leaving any space for passage of air. After removal of the plastic piece, the condition of the infant improved significantly. The child was switched back to conventional ventilator requiring less respiratory support.

## 3. Discussion

**Figure 1:**
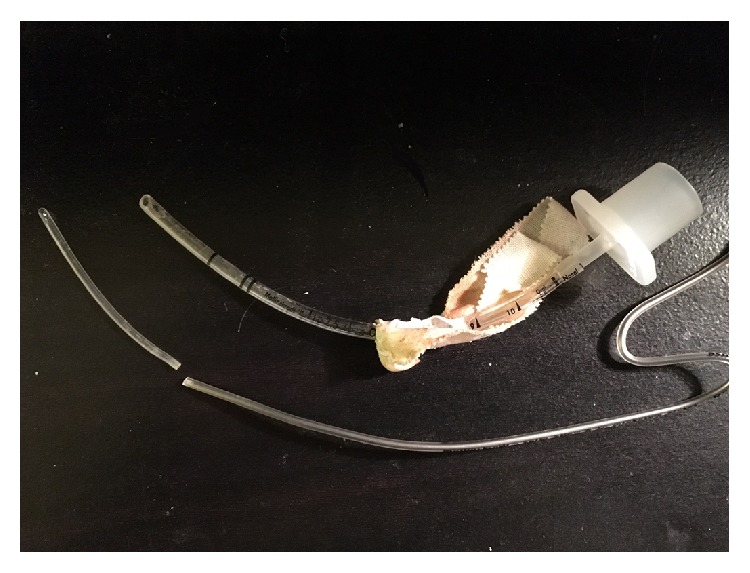
The sheared off piece of plastic and the stylet alongside the endotracheal tube.

**Figure 2:**
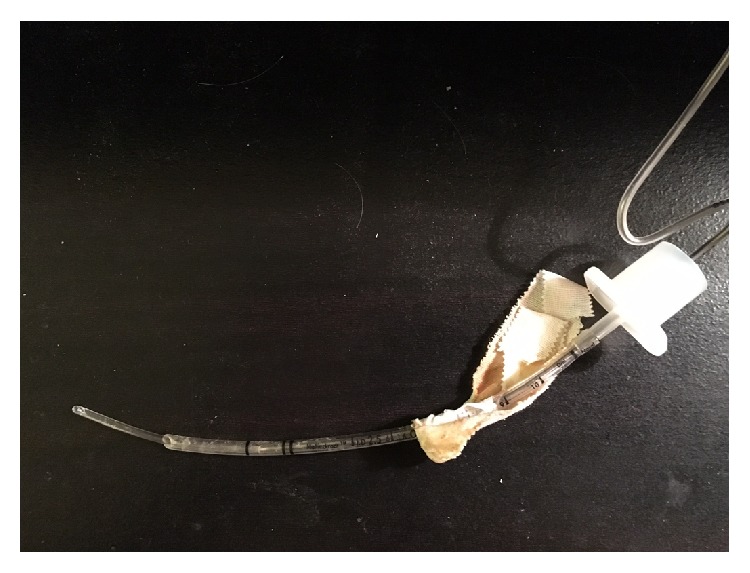
The orientation of the stylet along with the piece of plastic within the endotracheal tube, at the time of intubation.

In the past, shearing of the stylet has been reported to happen in 2.5 mm ETTs [1, 2]. Cook and Schultetus reported a case of a 700-gram newborn that was intubated soon after birth due to depressed respiration. They argued that due to a difference of only 0.5 mm between the diameter of the stylet and the internal diameter of the ETT the stylet gets tightly fitted within the lumen of the ETT. The tight fit and the soft plastic coating prevents the stylet from being removed easily from the ETT. Holding the ETT tightly by the clinician to prevent dislodgement also contributes to difficulty in removing the stylet leading to the pliable soft plastic coating being sheared from the tip of the stylet [1]. In our case, the plastic tip of the stylet was tightly lodged into the inner surface of the tip of the ETT. On application of excessive force by the clinician, this plastic sheath sheared off and remained within the inner diameter of the ETT partially obstructing the airway. When the stylet was pulled out, the plastic sheath over the aluminum surface of the stylet was visible till the tip of the stylet, giving the impression that the sheath was intact (Figure 1), although the broken tip was still in the ETT (Figure 2).

Another case was reported by Bhargava et al., where the plastic tip of the stylet was sheared off and remained in the ETT in a 1-month-old infant scheduled for bilateral inguinal hernia repair. Fortunately, in this case the missing distal part of the plastic coating of the stylet was immediately noticed upon removal of the stylet [3].

Chalhoub et al. reported a case of two large plastic fragments detached from a stylet while intubating a 27-year-old trauma patient. One piece was removed from the endotracheal tube a few hours later in the operating room while the other migrated asymptomatically into the pulmonary airway and was retrieved from the right bronchus 24 hour later. Fortunately, in our case, this serious complication did not occur [4].

## 4. Conclusion

This case demonstrates that application of excessive force while taking the stylet out after intubation could lead to shearing and lodging of the plastic sheath in the lumen of the ETT. Though this is a rare occurrence, this mechanism of mechanical obstruction of the ETT should be kept in mind while taking care of intubated infants in the NICU.

## Abbreviations

ETT: Endotracheal tube
NICU: Neonatal Intensive Care Unit.
